# RNAi knockdown of C-erbB2 expression inhibits salivary gland adenoid cystic carcinoma SACC-83 cell growth *in vitro*

**DOI:** 10.1016/S1674-8301(10)60031-0

**Published:** 2010-05

**Authors:** Xiaohua Liu, Yincheng Zhang, Wenhao Ren, Tengteng Cao, Yongjin Zhu

**Affiliations:** a Department of Oral and Maxillofacial Surgery, Xi'an Jiaotong University Stomatology Hospital, Xi'an 710004, Shaanxi Province, China; b Department of Center Research, Xi'an Jiaotong University Stomatology Hospital, Xi'an 710004, Shaanxi Province, China

**Keywords:** salivary gland adenoid cystic carcinoma, RNA interference, C-erbB2, gene silence

## Abstract

**Objective:**

To knockdown the C-erbB2 gene in salivary gland adenoid cystic carcinoma SACC-83 cells using RNA interference, and determine the effect of silencing C-erbB2 on cell proliferation.

**Methods:**

C-erbB2-siRNA was transfected into SACC-83 cells. RT-PCR and immunohistochemistry were used to detect C-erbB2 expression in SACC-83 cells. Cell proliferation was measured by the MTT assay and gene knockdown was achieved by RNA interference. Apoptosis was analyzed by flow cytometry.

**Results:**

Compared with the control, C-erbB2 mRNA expression was decreased in the C-erbB2-siRNA transfection group, and immunohistochemical analysis indicated that C-erbB2 protein expression was decreased. After C-erbB2-siRNA was transfected for 48 h, absorbance at 570 nm (MTT) was 0.185±0.021 compared with 0.354±0.034, 0.299±0.053, and 0.314±0.049 in the blank control, liposome control and negative control siRNA groups, respectively. The differences were statistically significant (*P* < 0.05) between the C-erbB2-siRNA group and the control groups. Following the C-erbB2 knockdown, the percentage of apoptotic cells was 5.63% compared with 2.04%, 2.85%, and 2.98% in the three control groups, respectively. Proliferation of SACC-83 cells was inhibited, and early apoptotic cells were increased.

**Conclusion:**

RNA interference can effectively silence C-erbB2 gene expression and inhibit growth of SACC-83 cells, which indicates the potential of targeting this gene as a novel gene therapy approach for the treatment of salivary gland adenoid cystic carcinoma.

## INTRODUCTION

Salivary gland adenoid cystic carcinoma (SACC) is one of the most biologically destructive and unpredictable of the oral and maxillofacial tumours and it is extremely difficult to treat[1]–[5]. Clinically, current therapies of SACC are surgery, chemotherapy and radiotherapy[6]–[8]. Gene therapy is a subject of current studies of potential tumour biology therapy[9]–[13]. The study of oncogene expression and function is fundamental for tumour therapy. In an earlier study, it was shown that the C-erbB2 gene was over-expressed in many types of tumours. The previous studies also confirmed that C-erbB2 was over-expressed in salivary gland cystic carcinoma[14]–[16]. In the present study, we used RNA interference (RNAi) to knockdown the C-erbB2 gene in the SACC-83 cell line, and observed the effect on cell growth of silencing C-erbB2.

## MATERIALS AND METHODS

### Cell culture

The SACC-83 cell line was obtained from the Peking University School of Stomatology. SACC-83 cells were cultured at 37°C in RPMI1640 medium containing 10% (v/v) calf serum, in a humidified 5% CO_2_ atmosphere. After confluence reached about 80% the cultured cells were digested by 0.25% (w/v) trypsin and subcultured.

### Design target sequence of siRNA

According to the principle of siRNA design[17]–[24], siRNA target sequences are usually 21 nt long, with 19 nt duplex and a 2 nt overhang toward the 3′ terminus, with AA or NA bases at the 5′-end of their targets, and a G+C content of 30–70%, provides the necessary thermodynamic stability for the siRNA. The target sequence should preferably not be very close to the initiation codon and should avoid regions within 75–100 bp of the start codon and the termination codon, because those regions are usually occupied by different protein factors. The target sequence should also avoid: intron regions, stretches of four or more base repeats (*e.g*., AAAA or CCCC), and both 5′ and 3′ untranslated regions. A BLAST homology search should be used to avoid sequences that share a certain degree of homology with other related or unrelated genes. It is important to design a negative control siRNA sequence, and to ensure that the control siRNA is not homologous to other genes. We considered the principle of siRNA design and we surveyed the literature to aid the design of siRNA target sequences of C-erbB2[25], which were named C-erbB2-siRNA, control-siRNA and FAM control-siRNA. The sense and antisense sequences of these siRNAs were, respectively: C-erbB2-siRNA 5′-GCCUCACAGAGAUCUUGAATT-3′, 5′-UUCAAGAUCUCUGUGAGGCTT-3′; control-siRNA 5′'-UUCUCCGAACGUGUCACGUTT-3′, 5′-ACGUGACACGUUCGGAGAATT-3′; FAM control-siRNA 5′-UUCUCCGAACGUGUCACGUTT-3′, 5′-ACGUGACACGUUCGGAGAATT-3′.

### Cell transfection

The following groups were designed: blank control, liposome control, negative control-siRNA and C-erbB2-siRNA. SACC-83 cells were seeded in six-well plates in 2 ml of growth medium without antibiotics for 24 h before transfection. When cells reached a confluence of 50–70%, they were transfected into siRNA with Lipofectamine 2000 (Invitrogen, USA). siRNA-Lipofectamine 2000 complexes were prepared for each transfection sample according to the manufacturer's protocol. The final concentration of siRNA when added to the cells was 50 nM. The siRNA-Lipofectamine 2000 complexes were added to each well containing cells and medium to a final volume of 2 ml. Cells were incubated at 37°C in a CO_2_ incubator until the assay for gene knockdown. The medium was replaced by complete medium after 6 h. Transfection efficiency was assayed by fluorescence microscopy after FAM control-siRNA was transfected for 6 h, and C-erbB2 expression was assayed after C-erbB2-siRNA was transfected for 48 h.

### RNA extraction and RT-PCR

Gene amplification primers were designed according to GenBank and published work[25]. The primers of C-erbB2 sense and antisense were, respectively: 5′-CTGTGCCCGAGTGTGCTA-3′, and 5′-GTCCCCATCAAAGCTCTCC-3′, and the product length was 142 bp. As a housekeeping gene, the primers of GAPDH sense and antisense were, respectively: 5′-GCACCGTCAAGGCTGAGAAC-3′, and 5′-TGGTGAAGACGCCAGTGGA-3′, and the product length was 138 bp.

At 48 h after transfection, total RNA was extracted from the cultured SACC-83 cells by TRIzol® Reagent (Invitrogen, USA) according to the manufacturer's instructions. RNA concentration and quality were assessed by measuring absorbance at wavelengths of 260 nm and 280 nm; *A*_260_/*A*_280_ was in the range 1.8–2.1. For the synthesis of single-stranded cDNA, random hexamer primers and RevertAid^TM^ M-MuLV reverse transcriptase (Fermentas Life Sciences, Canada) were used with 2.0 µg of total RNA from each sample. The reaction was done according to the manufacturer's instructions. PCR was done by standard method with the following PCR working system: cDNA template, 2 µl; forward primer, 1 µl; reverse primer, 1 µl; 2× *Taq* PCR MasterMix, 25 µl; and distilled water to a final volume of 50 µl. PCR conditions were: 94°C for 5min, followed by 30 cycles of 94°C for 30 s; 59°C for 30 s; and 72°C for 30 s; and finally 72°C for 7 min to terminate the reaction (20 cycles were used for GAPDH PCR). The PCR products were analyzed by electrophoresis in a 2% (w/v) agarose gel and visualized by staining with ethidium bromide to identify the amplified product of the expected size. All results were confirmed in five independent PCRs. For the semiquantification, an image of the gel was captured, and the intensity of each band was quantified using GeneTools from the Syngene gel analysis system.

### Immunohistochemical analysis

The SACC-83 cells were seeded on slides, and slides with equal cell distributions were selected for immunohistochemistry. The anti-C-erbB2 antibody was a rabbit polyclonal, and the secondary antibody was goat anti-rabbit (Beijing Boisynthesis Biotechnology Co., Ltd., China). Cells were fixed for 30 min with 4% (v/v) formaldehyde, rinsed in water, rinsed in PBS for 5 min, and then incubated at room temperature for 10 min in 3% H_2_O_2_ to block the activity of endogenous peroxidase. Normal goat serum was added and the seeded slides were kept at room temperature for 5 min to block non-specific protein and then incubated at 4°C with anti-C-erbB2 antibody overnight. On the following day, the slides were washed in PBS and kept at room temperature with a secondary antibody for 10 min, incubated with the S-A/HRP for 10 min, washed in PBS, and stained with DAB. The stain intensity, a marker for immunoreactivity, was assessed with a light microscope.

### MTT assay

The 3-(4,5-dimethylthiazol-2-yl)-2,5-diphenyltetrazolium bromide (MTT) assay was used for the assessment of cell proliferation. The test is based on the ability of live cells to cleave the tetrazolium ring in active mitochondria to a product molecule that absorbs light at a wavelength of 570 nm. SACC-83 were seeded on 96-well plates in 200 µl of medium for each well, cultured at 37°C for 24 h, and then siRNAs were transfected into the cells. Then the cells were cultured for 24 h, 48 h, 72 h or 96 h. Before each time point, 20 µl of MTT was added to each well followed by incubation at 37°C for 4 h. After removal of the medium, 200 µl of dimethylsulfoxide (DMSO) was added to each well. After gentle shaking, the absorbance at 570 nm was measured.

### Flow cytometric analysis of apoptosis

The apoptosis of cells was detected with an Annexin-V-FITC apoptosis detection kit (Abcam Technical Antibody, UK). Cells were transfected, and culturing was continued for 48 h. Cells were then collected and suspended in 500 µl of binding buffer, and 2 µl of Annexin-V-FITC and 5 µl of propidium iodide (PI) were added. The apoptosis analysis was performed with a flow cytometer. The sample was incubated for 5 min in the dark before analysis.

### Statistical analysis

Data are expressed as mean±SD. All statistical analyses used SPSS software (Version 13.0, SPSS Inc., USA). Comparison of variables between groups was done by One-Way ANOVA or the non-parametric Kruskal-Wallis test, as appropriate. ANOVA of Repeated Measures was performed in the MTT assay. *P* < 0.05 was set as the level of statistical significance.

## RESULTS

### mRNA expression of C-erbB2

**Fig. 1:**
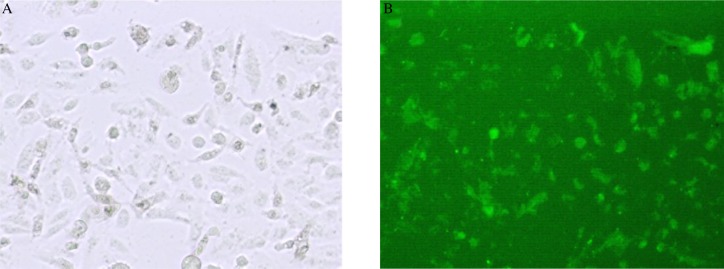
Transfection efficiency of siRNA. FAM control siRNA transfected SACC-83 cell in light microscope field (A, ×400) and fluorescent microscope field (B, ×400).

**Fig. 2:**
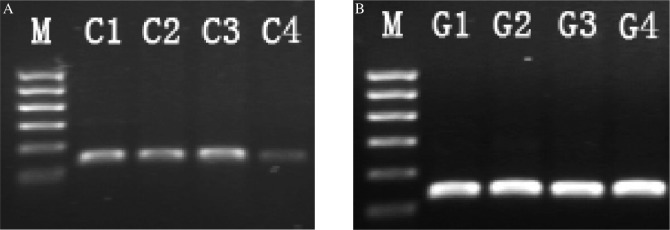
PCR products electrophoresis. A: The mRNA expression of C-erbB2 in the blank control group (C1), the liposome control group (C2), the negative control-siRNA group (C3), and the C-erbB2-siRNA group (C4), respectively. B: The mRNA expression of GAPDH in the blank control group (G1), the liposome control group (G2), the negative control-siRNA group (G3), and the C-erbB2-siRNA group (G4), respectively. M lane is DNA marker 100 bp-600 bp.

After FAM control-siRNA was transfected for 6 h, we assayed transfection efficiency by fluorescence microscopy. The siRNA was transfected into SACC-83 cells with a transfection efficiency of 87% (***Fig. 1***). Total RNA was analyzed by electrophoresis in 2% agarose gel. There was one band at the positions equivalent to 28 S, 18 S, and 5 S, and the intensity of the 28 S band was double that of the 18 S band. The C-erbB2 PCR products were subjected to electrophoresis and the length of the PCR amplification product was the same as the expected size. The results of the electrophoresis indicated that C-erbB2 mRNA was decreased in the C-erbB2-siRNA transfected group, lower than that in the blank control group, the liposome control group and the negative control-siRNA group (***Fig. 2A***), and the difference was significant, (*P* < 0.05). The RNAi knockdown expression of C-erbB2 mRNA in SACC-83 was successful. The expression of C-erbB2 mRNA was not significantly different among the control groups (*P* > 0.05).

### Protein expression of C-erbB2

**Fig. 3:**
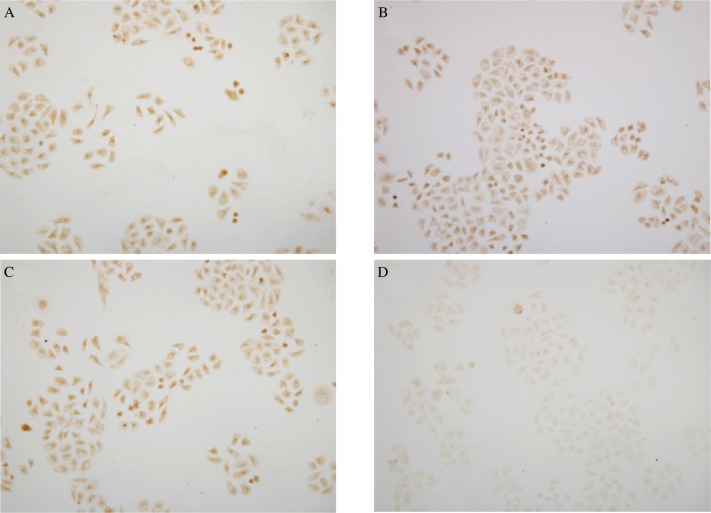
The protein expression of C-erbB2 (Immunohistochemistry ×200). A: the blank control group. B: the liposome control group. C: the negative control-siRNA group. D: and the C-erbB2-siRNA group.

Immunohistochemical staining showed that the protein of C-erbB2 was localized on cell membranes, in agreement with the results of other studies. Immunohistochemical images were analyzed by Image-ProPlus software. Under the same conditions, the Gray-scale of the stained area was detected. The average absorbance value of C-erbB2 protein expression in the blank control, the liposome control and the negative control-siRNA groups was 0.060±0.013 (*n* = 5), 0.068±0.019 (*n* = 5) and 0.068±0.012 (*n* = 5), respectively, but the average absorbance value of C-erbB2 protein expression in the C-erbB2-siRNA transfection group was 0.019±0.008 (*n* = 5). The results of immunohistochemistry indicated that the level of C-erbB2 protein was decreased significantly in the C-erbB2-siRNA transfection group, lower than that in the blank control, liposome control and negative control-siRNA groups (***Fig. 3***), and the difference was significant (*P* < 0.05).

### C-erbB2 RNAi inhibited proliferation of SACC-83 cells

**Fig. 4:**
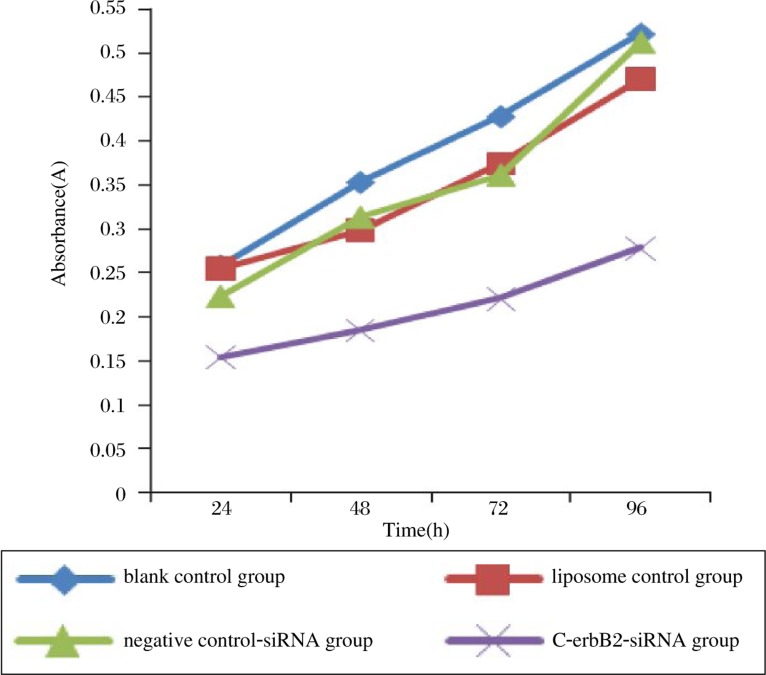
The cell growth curve assessed by MTT assay.

The effect of C-erbB2 silencing on cell proliferation was measured by the MTT assay. At 48 h, the absorbance in the blank control, the liposome control and the negative control-siRNA groups was 0.354±0.034 (*n* = 5), 0.299±0.053 (*n* = 5) and 0.314±0.049 (*n* = 5), respectively; and the absorbance in the C-erbB2-siRNA transfected group was 0.185±0.021 (*n* = 5). The proliferation of C-erbB2-siRNA infected cells was significantly slower than that of the control groups (*P* < 0.05) (***Fig. 4***).

### C-erbB2 RNAi enhanced apoptosis in SACC-83 cells

**Fig. 5:**
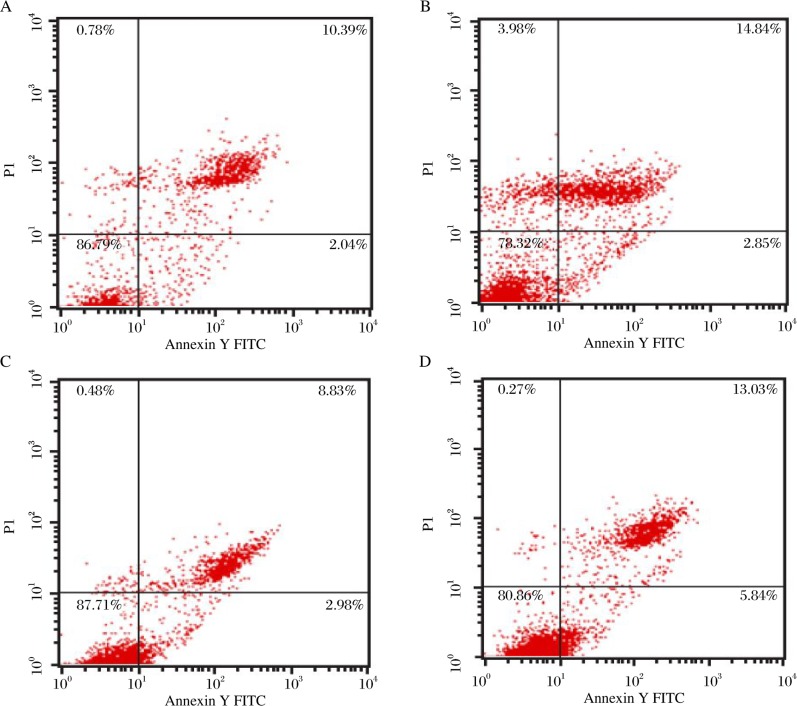
Cell apoptosis by flaw cytometic analysis. A: the blank control group. B: the liposome control group. C: the negative control-siRNA group. D: the C-erbB2-siRNA group.

After C-erbB2 was silenced by RNAi, cell apoptosis was measured by flow cytometry. We found that apoptosis was different between the C-erbB2 RNAi group and the control groups. Following the C-erbB2 gene knockdown, the early apoptosis of cells was 5.63% and that of the control groups was 2.04%, 2.85% and 2.98%, respectively (***Fig. 5***). The results showed that C-erbB2 silencing induced SACC-83 cell apoptosis.

## DISCUSSION

Malignant tumours pose serious hazards to human health. Adenoid cystic carcinoma is a malignant neoplasm of the salivary glands. The behavior of SACC has been shown to be unpredictable in terms of both distant and local spread. The tumour which has a significant propensity for perineural spread and distant metastasis is associated with a high mortality rate and often recurs after prolonged periods of time.

The C-erbB2 proto-oncogene is a member of the human epidermal growth factor (EGF) family. It is a member of the cell surface receptor tyrosine kinases (RTKs), which can trigger a multitude of signaling pathways, such as MAPKs and PI3K, within the cell and are active as homo- or heterodimers. C-erbB2 proto-oncogene encodes the p185 transmembrane protein. Oncogenic activation by C-erbB2 can occur through its over-expression, point mutation within the transmembrane domain or deletion of the extracellular domain. C-erbB2 activates downstream signaling pathways that regulate cell proliferation, survival, differentiation and transformation. C-erbB2 plays an important role in carcinogenesis and C-erbB2 gene dysfunction can induce tumour formation. Over-expression of the C-erbB2 gene has been observed frequently in human tumours, including those of the breast, ovary, lung, stomach, and oral cavity[26]–[31].

Gene silencing can be achieved in mammalian cells by the introduction of siRNAs, which mimic the initial step of the RNAi mechanism and the siRNAs can be used to study specific gene functions[32]–[34]. Kim *et al*. studied the relationship of ErbB2 and MIC-1 using siRNA downregulation of ErbB2. The results showed that MIC-1 might participate in the malignant progression of human breast and gastric cancer cells that over-express ErbB2 through the transactivation of ErbB2 tyrosine kinase[35]. Jiang *et al*. silenced C-erbB2/HER2 in 16HB4-T cells using short hairpin RNA (shRNA). Silencing of C-erbB2/HER2 resulted in significant increase in the proportion of cells in the G0/G1 phase, decrease in the proportion of cells in the S phase, and reduced cell viability[36]. In an earlier study, the data confirmed that C-erbB2 was over-expressed in SACC[14],[15],[37]; so our aim was to knockdown the C-erbB2 gene in SACC using RNAi. In this study, after C-erbB2-siRNA was transfected into adenoid cystic carcinoma cells, the expression of C-erbB2 was decreased significantly at both the mRNA and the protein levels in the SACC-83 cells compared to controls. Apoptosis is an important physiological function of the cell. Cancer is a malignant hyperplastic disease, and the balance between proliferation and apoptosis is dysregulated in cancer cells. Gene abnormalities such as mutation or amplification are detected frequently during the course of cancer development, which might be related to cancer progression. We used the MTT assay to observe the effect of silencing C-erbB2 on cells and found that SACC cells exhibited lower viability after C-erbB2 knockdown. Flow cytometry analysis of apoptosis showed that early apoptosis of cells was enhanced. The results indicate that inhibition of C-erbB2 expression could reduce SACC cell growth.

In conclusion, C-erbB2 was effectively knocked down in SACC-83 cells by RNA interference, and apoptosis of cancer cells was enhanced and cell growth was inhibited. These results also suggested that the C-erbB2 gene has an important role in salivary gland adenoid cystic carcinoma. Results of the present study also suggest that C-erbB2 is a feasible RNAi target gene for therapy against salivary gland adenoid cystic carcinoma.
